# High-Temperature-Resistant Fiber Laser Vector Accelerometer Based on a Self-Compensated Multicore Fiber Bragg Grating

**DOI:** 10.3390/s22176459

**Published:** 2022-08-27

**Authors:** Xunzhou Xiao, Jun He, Xizhen Xu, Runxiao Chen, Bin Du, Yanping Chen, Shen Liu, Cailing Fu, Yiping Wang

**Affiliations:** 1 Key Laboratory of Optoelectronic Devices and Systems of Ministry of Education/Guangdong Province, College of Physics and Optoelectronic Engineering, Shenzhen University, Shenzhen 518060, China; 2 Shenzhen Key Laboratory of Photonic Devices and Sensing Systems for Internet of Things, Guangdong and Hong Kong Joint Research Centre for Optical Fibre Sensors, Shenzhen University, Shenzhen 518060, China

**Keywords:** fiber Bragg gratings, multicore fiber, femtosecond laser, vector accelerometer

## Abstract

We propose and demonstrate a novel high-temperature-resistant vector accelerometer, consisting of a ring cavity laser and sensing probe (i.e., fiber Bragg gratings (FBGs)) inscribed in a seven-core fiber (SCF) by using the femtosecond laser direct writing technique. A ring cavity laser serves as a light source. Three FBGs in the outer cores of SCF, which are not aligned in a straight line, are employed to test the vibration. These three FBGs have 120° angular separation in the SCF, and hence, vibration orientation and acceleration can be measured simultaneously. Moreover, the FBG in the central core was used as a reflector in the ring cavity laser, benefiting to resist external interference factors, such as temperature and strain fluctuation. Such a proposed accelerometer exhibits a working frequency bandwidth ranging from 4 to 68 Hz, a maximum sensitivity of 54.2 mV/g, and the best azimuthal angle accuracy of 0.21° over a range of 0–360°. Furthermore, we investigated the effect of strain and temperature on the performance of this sensor. The signal-to-noise ratio (SNR) only exhibits a fluctuation of ~1 dB in the range (0, 2289 με) and (50 °C, 1050 °C). Hence, such a vector accelerometer can operate in harsh environments, such as in aerospace and a nuclear reactor.

## 1. Introduction

Accelerometers (vibration sensors) have been applied in many fields, such as structural health monitoring [1,2], geological disaster early warning [3,4], and oil exploration [5,6]. Such an accelerometer is crucial to identify the stage of damage before catastrophic failure and prevent potential safety issues. For example, accumulated damage in turbine blades could be diagnosed by monitoring the changes in vibration frequency [7]. A life-saving early earthquake warning could be given by monitoring the arrival of the P wave [3]. Compared with conventional piezoelectric sensors and micro-electro-mechanical Systems (MEMS) [2], optical fiber sensors exhibit unique advantages [8] (e.g., minimal size, resistance to high temperature, immunity to electromagnetic interference, and long-distance detection), becoming perfect candidates to achieve vibration measurement in extremely harsh environments [9,10].

In recent years, optical fiber accelerometers based on the Fabry–Perot interferometer (FPI) and fiber Bragg grating (FBG) have been proposed and demonstrated [11,12,13,14,15,16,17]. FPI-based accelerometers are highly sensitive and easy to fabricate. Moreover, such sensors created on a sapphire fiber can operate at a high temperature of 800 °C [12]. However, they can merely measure acceleration in a single direction, where it is hard to distinguish the actual magnitude and orientation, especially when the vibration source is unknown. Additionally, they require at least two orthogonal FPI accelerometers to realize vector vibration measurement, which makes the system complex and bulky [18]. 

To address this issue, FBG bundle accelerometers were proposed. By parallelly integrating multiple FBGs in a fixed geometry configuration, the azimuthal angle and magnitude of vibrational acceleration can be calculated [19]. However, the size of the FBG bundle is still too large to be applied in some narrow environments, such as slim holes, aero-engine pipelines, and reactor fuel pins. Fortunately, tilted FBG, cladding FBG, and eccentric FBG could realize vector vibration sensing only by using a single FBG since the cladding mode resonance is orientation sensitive [20,21,22]. Hence, the size of accelerometers based on the above-mentioned FBGs is merely hundreds of micrometers. However, the unwanted power fluctuations in cladding modes, introduced by the change in surrounding refractive index (SRI), would perturb the vibration response. Multicore fiber Bragg grating (MC-FBGs) accelerometers have been demonstrated to solve this problem [23], since the vibration elements (i.e., the FBGs inscribed in the fiber cores) are not susceptible to external SRI. Moreover, the FBG inscribed in the central core can be used for temperature compensation. In most previous reports, MC-FBGs were fabricated by using a UV holographic method [24,25], limiting their potential application in high-temperature environments. Thanks to the MC-FBGs fabricated by using a femtosecond laser, compact vector high-temperature accelerometers are promising to achieve [26].

In this letter, we report for the first time, to the best of our knowledge, a novel high-temperature-resistant vector accelerometer based on a ring cavity laser and FBGs inscribed in a seven-core fiber (SCF) by using the femtosecond laser direct writing technique. Three FBGs in the outer cores act as vector vibration sensing elements. Compared with the present high-temperature-resistant fiber laser sensor [27,28], the use of a ring-cavity laser and SCF endows our sensor with the ability to compensate for temperature and strain fluctuation. The FBG in the central core was employed as a narrow band reflector in the ring cavity laser, enabling the laser to achieve the ability of temperature and strain compensation. The sensing performance of the proposed accelerometer, including frequency, acceleration sensitivity, orientation dependence, and thermal stability, was demonstrated and discussed. The results show that our proposed accelerometer can meet the requirement of vector vibration sensings in harsh environments, such as underground pipeline detection and oil well leak detection.

## 2. Principle of Acceleration and Orientation Measurement

**Figure 1:**
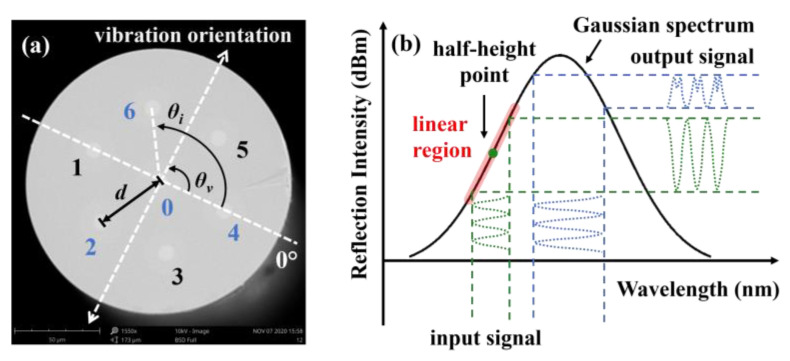
(**a**) Scanning electron microscopy image of the homogeneous SCF with the defined geometrical parameters and (**b**) schematic diagram of interrogation.

The SCF (YOFC) used to create the accelerometer includes a central core and six outer cores. As shown in Figure 1a, the diameters of the cores and cladding are ~8 and ~150 μm, and the core spacing d between two adjacent cores is ~42 μm. The principle of the FBG-based accelerometer is to monitor the Bragg wavelength shifts of the gratings inscribed in different cores during the period of vibration. During the vibration process, one of the outer cores is stretched, and the Bragg wavelength would exhibit a “red” shift. On the contrary, a blue shift occurs when the core is compressed. The Bragg wavelength shift (∆*λ*) can be expressed as:

(1) $$\Delta \lambda_{i}=\left(1-p_{\varepsilon }\right)\lambda_{i}\varepsilon_{i},$$

where *p_ε_* is the effective photoelastic coefficient, relative to the effective index of the core and Poisson’s ratio of the fiber, and *ε_i_* is the strain induced by the vibration on FBG in core *i*, which can be expressed as:

(2) $$\varepsilon_{i}=\frac{d_{i}}{R}\sin\left(\theta +\theta_{i}\right),$$

(3) $$\theta =\pi /2-\theta_{v},$$

where *d_i_* is the spacing between the outer core *i* (i.e., core 1–6) and core 0, as illustrated in Figure 1a. *θ_v_* and *θ_i_* indicate the orientation of the vibration and angular position of each outer core *i*, respectively. *R* is the vibration-induced bending radius.

In order to obtain the vibration orientation and acceleration simultaneously in a single measurement, at least two cores that are not in diametrically opposite positions need to be recorded simultaneously. Here, we chose outer cores 2, 4, and 6 to record the periodic change in strain during vibration. Since *R* is much larger than *d* and ∆*λ_i_* is much smaller than *λ_i_*, the vibration azimuthal angle *θ_v_* can be described as follows:

(4) $$\theta =\arctan\left(\frac{\sin\theta_{j}\Delta \lambda_{i}/\lambda_{i}-\sin\theta_{i}\Delta \lambda_{j}/\lambda_{j}}{\cos\theta_{i}\Delta \lambda_{j}/\lambda_{j}-\cos\theta_{j}\Delta \lambda_{i}/\lambda_{i}}\right),$$

where *i* and *j* represent two chosen cores with an angle of 2π/3 (i.e., *i* = 2 and *j* = 4). Once the orientation of the vibration is determined, using the measured sensitivities under varying orientations, together with the wavelength shifts of the corresponding fiber cores, the acceleration value can be identified. As a result, orientation and acceleration can be obtained simultaneously within a two-dimensional range. 

However, detecting wavelength shifts requires an expensive wavelength interrogation. To solve this issue, a ring cavity laser was embedded to monitor the Bragg wavelength shift. The spectral width of the laser is much narrower than that of the FBG; hence, the laser spectrum and FBG spectrum can be assumed as a delta function and an ideal Gaussian function. As shown in Figure 1b, the Gaussian spectrum has a linear region near the half-height point. By monitoring reflected power differences, the Bragg wavelength shift can be linearly tracked. By comparing the amplitude difference of the three outer cores and their relative positions, according to Equation (3), the vibration orientation *θ_v_* can be described as follows:

(5) $$\theta =\arctan\left(\frac{\sin\theta_{j}\Delta V_{i}/V_{i}-\sin\theta_{i}\Delta V_{j}/V_{j}}{\cos\theta_{i}\Delta V_{j}/V_{j}-\cos\theta_{j}\Delta V_{i}/V_{i}}\right),$$

where *V* is the amplitude of the reflected power, and *i* and *j* are two chosen cores with an angle of 2π/3 (i.e., *i* = 2 and *j* = 4). To consistently track the half-height point and ensure that the input signal is located in the linear region at all moments, the FBG in the central core (i.e., core 0) was used as a reflector in the ring cavity laser. Since the transversal position of the central core (i.e., core 0) is located on the strain neutral plane at all times, leading to the vibration insensitivity of FBG in core 0, the laser wavelength and output remain unchanged. Such a character endows the laser with a self-compensated ability, which can resist external interference factors, such as temperature and strain fluctuation, free from expensive wavelength interrogation and complex data processing algorithms for temperature and strain compensation.

## 3. Experimental Setup and Results

**Figure 2:**
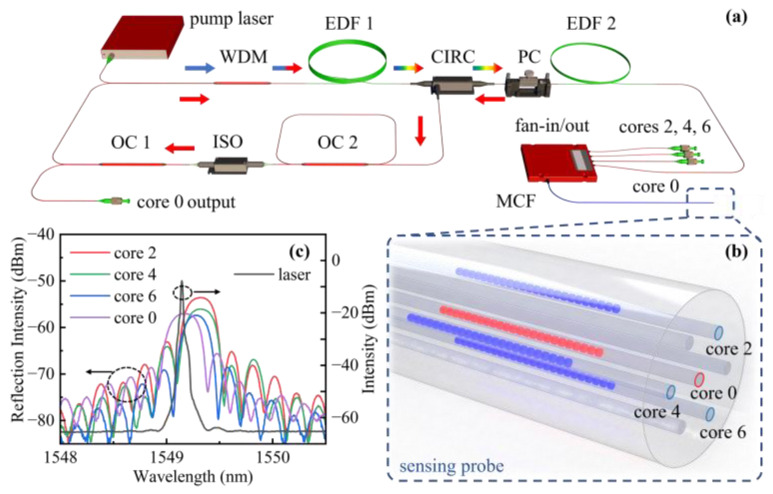
(**a**) Experimental setup for the ring cavity laser used for the interrogation of the two-dimensional accelerometer, (**b**) distribution of FBGs in SCF (i.e., sensing probe), (**c**) reflection spectra of the FBGs inscribed in the fiber cores and the output spectra of the laser.

The proposed two-dimensional accelerometer consists of a ring cavity laser and sensing probe (i.e., SCF-FBGs). As shown in Figure 2a, a 6 m long erbium-doped fiber ( nLIGHT, Camas, WA, USA, Liekki Er16-8/125, peak absorption: 16 dB/m @ 1530 nm,) (i.e., EDF1) is used as a gain medium, which is pumped by a 1480 nm laser diode through the wavelength division multiplexer (WDM). The FBG in the central core (i.e., core 0) of an SCF is used as the coarse wavelength selection element, which is connected to the ring cavity through an optical circulator and fan-in/out (YOFC, insertion loss: less than 2 dB). Moreover, a 50:50 optical coupler (OC1) is used to determine the ratio between the injection power and output laser. The isolator (ISO) is employed to ensure that the laser propagates in a single direction. Hence, a typical multiple longitudinal mode fiber ring laser with high-power output is achieved. This results in a high signal-to-noise ratio (SNR), which is conducive to sensing applications. Moreover, an additional 2 m unpumped EDF2 and a polarization controller (PC) were applied as an adjustable polarized saturable absorber, which is used to reduce the longitudinal mode density [29]. Furthermore, a secondary cavity composed of a 50:50 OC2 was inserted into the main cavity to expand the mode spacing owing to the Vernier effect [30] and eventually achieve a single longitudinal mode operation, avoiding the unwanted wavelength and output power fluctuation. The optical module of the laser is packaged in a metal cavity to insulate the device from external vibration. All the open pigtail fibers are cleaved at 8° to suppress the reflections of the end facets.

In this work, we used a femtosecond laser auto-positioning point-by-point technology to fabricate FBGs in the SCF, and the experimental setup can be found in our previous work [31]. A frequency-doubled regeneratively amplified Yb: KGW femtosecond laser (Light Conversion, Vilnius, Lithuania, Pharos) with a pulse width of 290 fs, and a repetition rate of 200 kHz was employed. The femtosecond laser beam with a pulse energy of 42 nJ was focused into the core center through the fiber coating, creating a series of index modulation regions with a fixed interval, thus forming an FBG. As shown in Figure 2b, four FBGs were created in cores 0, 2, 4, and 6 at the same cross section of the SCF, and all of them have the same grating length of 4 mm. Note that the periods of three FBGs in outer cores and the FBG in the central core are 1.0824 and 1.0822 μm, respectively. The SCF was spliced with a fan-in/out device using a fusion splicer (Fujikura, Tokyo, Japan, FSM-100P+), and the splice loss was less than 0.7 dB. Reflection spectra were measured using a broadband light source with a wavelength range of 1250 to 1650 nm (Fiber Lake, Hulbert, OK, USA, ASE-LIGHT SOURCE) and an optical spectrum analyzer with a resolution of 0.02 nm. (Yokogawa, Tokyo, Japan, AQ6370D). As shown in Figure 2c, the Bragg wavelengths of four FBGs inscribed in cores 0, 2, 4, and 6 were 1564.81, 1564.98, 1564.91, and 1565.06 nm, respectively. The full width at half maximum (FWHM) of FBGs is ~0.3 nm. They were employed as two-dimensional vibration sensing elements. The reflectivities in these FBGs range from 51.13% and 54.71%, providing enough feedback to form a resonant cavity and being suitable for intensity interrogation in subsequent vibration sensing experiments. Furthermore, the FBG in core 0 was used as a reflector in the ring cavity laser, exhibiting a lasing wavelength of ~1564.82 nm and an SNR of 64.5 dB at a pump power of 200 mW. Here, such a ring cavity laser serves as a light source, with the wavelength designed to be 0.2 nm less than the Bragg wavelength of FBGs in outer cores (near the half-height point of the reflection spectrum), resulting in the laser wavelength aligned in the linear region of the FBGs in outer cores. As a result, the wavelength shifts of probe FBGs were converted to intensity variation linearly. Although the difference in reflectivities may cause reflection intensity difference, it can be eliminated by calibration under a fixed acceleration.

**Figure 3:**
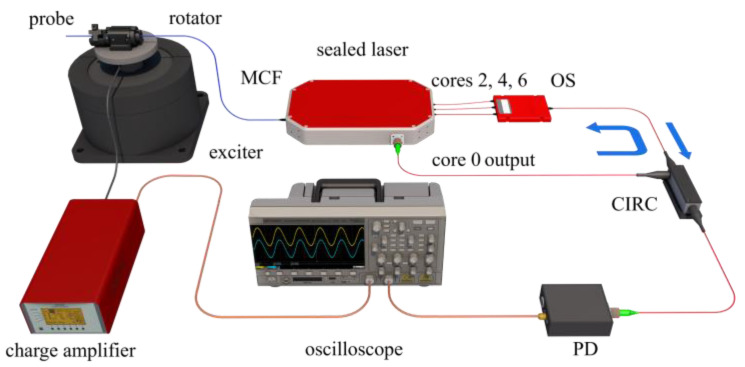
Schematic diagram of the experimental setup for the vector vibration detection system.

The experimental setup of the vector vibration detection system is illustrated in Figure 3. The sensing probe was fixed on a fiber rotator (Thorlabs, Newton, NJ, USA, HFR007) with an initial orientation angle of 350°, and both of them were mounted on an exciter (Bruel & Kjaer, Nerum, Denmark, 4808) driven by a sinusoidal signal generator. Note that a section of free fiber with a length of 35 mm downstream from gratings behaves as an inertial mass, which determines the resonance frequency. The output of the ring cavity laser was reflected by the FBGs in outer cores through the circulator, optical switch, and fan-in/out. The reflected power change was measured by using a photodetector (PD, 2053 Newport). Moreover, a referential accelerometer was vertically fastened to the exciter and amplified by a charge amplifier to characterize its performance. Then, signals from the PD and charge amplifier were recorded, processed, and displayed on an oscilloscope (Tektronix, Beaverton, OR, USA, MDO3054).

**Figure 4:**
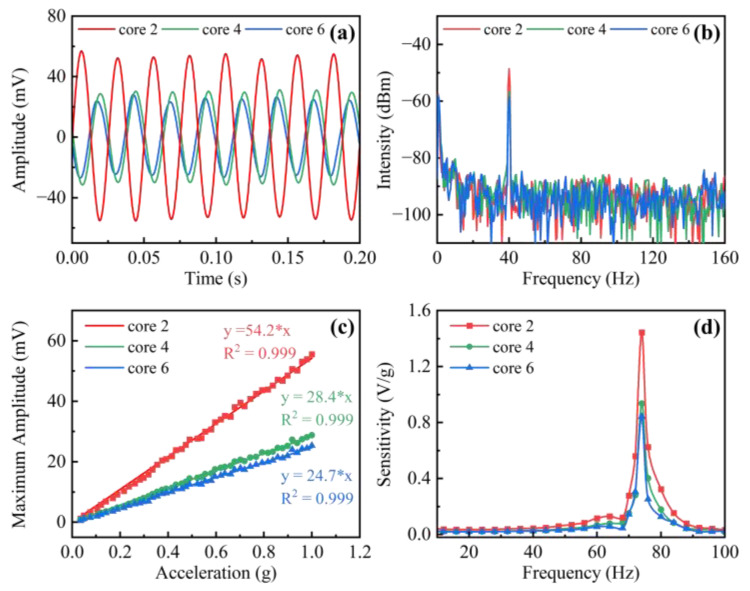
(**a**) Real-time reflected power and (**b**) the corresponding FFT spectrum in cores 2, 4, and 6 with an exciting frequency of 40 Hz and an acceleration of 1.0 g. (**c**) Reflected power versus applied acceleration ranging from 0.03 to 1.0 g; (**d**) sensitivity-frequency responses of the FBGs in cores 2, 4, and 6 with an acceleration of 1.0 g under an orientation angle of 350°.

Subsequently, the vibration test was carried out on the fabricated two-dimensional accelerometer. A sine vibration wave with a frequency of 40 Hz and an acceleration of 1.0 g (g = 9.8 m/s^2^) was applied to the sensing probe. As shown in Figure 4a, curves present the amplitude changes of the FBGs in cores 2, 4, and 6 in the time domain. The corresponding maximum amplitudes of these cores are different, which could be used to distinguish the vibration orientation. Figure 4b demonstrates the fast Fourier-transform (FFT) spectra of the time-domain results, and it can be seen that the measured frequency agrees well with the exciting frequency from the signal generator. Furthermore, the acceleration was increased from 0.03 to 1.0 g, as displayed in Figure 4c, the maximum amplitude of three cores exhibits a linear response. The sensitivities of the FBGs in cores 2, 4, and 6 are 54.2, 28.4, and 24.7 mV/g, respectively. Additionally, the frequency characteristics of the sensing probe have been evaluated ranging from 4 to 100 Hz in steps of 4 Hz, while the acceleration remained constant at 1.0 g. As shown in Figure 4d, the corresponding sensitivity exhibits a maximum of 1.44 V/g at 74 Hz, which agrees well with the theoretical resonance frequency calculated as 72.3 Hz. The proposed accelerometer exhibits a flat response range at a lower frequency, which could be increased by attaching additional mass to the end of the free fiber or shortening the length of the free fiber [25].

**Figure 5:**
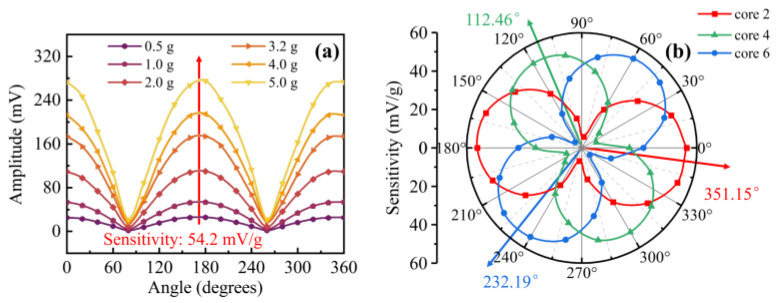
(**a**) Orientation response of core 2 at different accelerations ranging from 0.5 to 5.0 g; (**b**) acceleration sensitivities of the FBGs in the three outer cores 2, 4, and 6, plotted for various orientation angles in polar coordinate.

The capability of orientation discrimination for the proposed two-dimensional accelerometer was investigated by applying different accelerations with various orientations. In this experiment, an exciting frequency was set to 40 Hz. The reflected power of the FBG in core 2 was recorded with different orientation angles from 0° to 360° in steps of 30°, and repeated tests were performed under different acceleration amplitudes from 0.5 to 5.0 g. As shown in Figure 5a, all reflected powers for a given acceleration amplitude exhibited good agreement with the absolute value of sinusoidal curves (i.e., the responses of the reflected power to the accelerations were different in various orientation angles). Then, the linear fitting was adopted to obtain the sensitivities of the FBGs in cores 2, 4, and 6, and the results are plotted in the polar coordinate system in Figure 5b. There were three perfect ‘8’-shaped patterns corresponding to the three outer core FBGs, and each ‘8’-shaped pattern included two maxima and two minima. Moreover, the included angle between maximal sensitivity directions of cores 2, 4, and 6 is 120° (i.e., 351.15°, 232.19°, and 112.46° relative to the arbitrary 0°), which was consistent with their 120° angular separation in the SCF.

**Figure 6:**
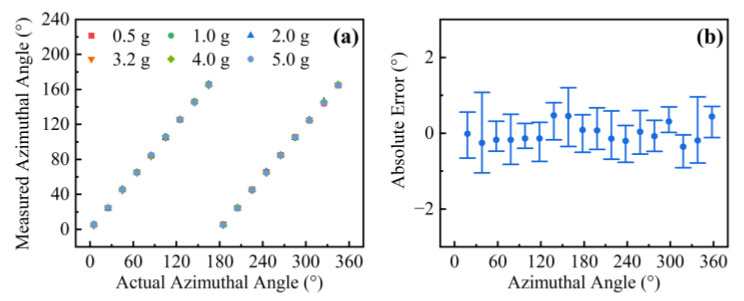
(**a**) Actual and measured orientation angle at various accelerations ranging from 0.5 to 5.0 g and (**b**) corresponding accuracy ranges.

Furthermore, we employed such an accelerometer to test an unknown vibration. The frequency can be easily obtained by conducting the FFT from the time-domain reflected power. Then, in order to extract the accelerations and orientations, we used Equation (4) to calculate the orientation angle *θ_v_*, and the accelerations can be acquired by referring to the calibrated sensitivities in Figure 5. Figure 6a shows the theoretical results of orientation *θ_v_* reconstructed from the measured reflected power at different input angles plotted against the measured azimuthal angles. Additionally, the various accelerations ranging from 0.5 to 5.0 g at certain orientations are characterized. It can be seen that the measured orientation values stay consistent with the actual values in the range of 0° to 180°. However, this sensor has the same vibration response at 0–180° and 180–360° since the fiber is circularly symmetric. As shown in Figure 6b, the orientation accuracy was represented by using an error bar, ranging from 0.21° to 1.53° at all orientation angles, which is close to that of the accelerometer in [25].

**Figure 7:**
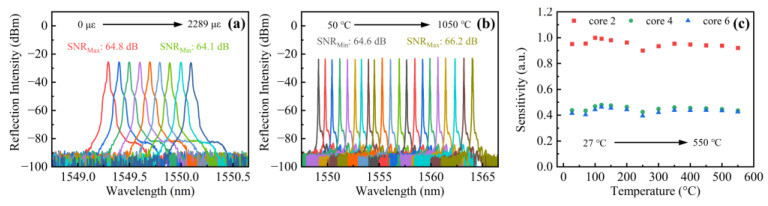
Evolution of reflection spectra of the accelerometer at (**a**) various strains ranging from 0 to 2289 με and (**b**) various temperatures ranging from 50 to 1050 °C. (**c**) Sensitivity-temperature responses of the accelerometer in the range of 27 to 550 °C with an exciting frequency of 40 Hz and an acceleration of 5.0 g.

We further investigated the temperature and strain responses of the fabricated accelerometer. In this experiment, a pump power of 200 mW was employed, and the output of the laser was reflected by the FBG in core 2, and a signal with an SNR of 64.8 dB can be obtained. The strain response of this device was tested by fixing one end of the sensor and stretching the other end fixed on a translation stage, ranging from 0 to 2289 με in steps of 254 με. The spectra were measured and are shown in Figure 7a. Note that the laser wavelength exhibits a “red” shift with an increasing strain, while the SNR only has a bit of fluctuation (i.e., 0.5 dB). Thus, this proposed accelerometer is free from the impact of strain. Furthermore, the accelerometer was placed in the furnace (Carbolite Gero, Derbyshire, UK, EHC12-450B), which has a uniform temperature field within a length of 90 mm and an accuracy of ±5 °C. The temperature in the furnace varied from room temperature to 1050 °C and was maintained for 30 min at each measurement point. An embedded N-type thermocouple was placed along the sample to record the temperature. As illustrated in Figure 7b, similarly, the laser wavelength exhibits a “red” shift with an increasing temperature. Additionally, the SNR exhibits a slightly larger fluctuation of ~2 dB. The reason for this phenomenon is that the residual stress relaxation would occur at high temperatures, resulting in the change in the spectra of FBGs. However, thanks to the type II grating structures induced by a femtosecond laser, our proposed accelerometer still has high-temperature resistance [32]. To further investigate the temperature stability of our proposed accelerometer, the vibration measurements were carried out in the range of 27 to 550 °C. Figure 7c displays the normalized sensitivity versus the temperature for cores 2, 4, and 6 when acceleration is 0.5 g and the exciting frequency is 40 Hz. The sensitivity only shows a small fluctuation of 10%, proving its stability and reliability in harsh environments.

## 4. Conclusions

We have proposed and demonstrated a high-temperature-resistant vector accelerometer based on a ring cavity laser and SCF-FBGs inscribed by using the femtosecond laser direct writing technique. A ring cavity laser surveys as a light source. Three FBGs in the outer cores have 120° angular separation. They were used to measure acceleration and vibration orientation simultaneously. Note that the FBG in the central core of an SCF was used as a reflector to construct the ring cavity laser, which could be used to achieve temperature and strain compensation. The proposed accelerometer exhibits a working frequency bandwidth ranging from 4 to 68 Hz, a maximum sensitivity of 54.2 mV/g, and the best azimuthal angle accuracy of 0.21° over a range of 0–360°. Moreover, the effects of strain and temperature on the performance of this device were investigated. The results show that the SNR of the sensor exhibits excellent stability at 0 to 2289 με and room temperature to 1050 °C. Therefore, such a vector accelerometer could be applied in extreme environments, such as in aerospace and a nuclear reactor.

## Data Availability

Not applicable.
